# Identification of microbial community in the urban environment: The concordance between conventional culture and nanopore 16S rRNA sequencing

**DOI:** 10.3389/fmicb.2023.1164632

**Published:** 2023-04-13

**Authors:** Annie Wing-Tung Lee, Chloe Toi-Mei Chan, Lily Lok-Yee Wong, Cheuk-Yi Yip, Wing-Tung Lui, Kai-Chun Cheng, Jake Siu-Lun Leung, Lam-Kwong Lee, Ivan Tak-Fai Wong, Timothy Ting-Leung Ng, Hiu-Yin Lao, Gilman Kit-Hang Siu

**Affiliations:** Department of Health Technology and Informatics, The Hong Kong Polytechnic University, Hong Kong, Hong Kong SAR, China

**Keywords:** urban microbiome, nanopore sequencing, 16S rRNA gene sequencing, taxonomic classifiers, conventional culture

## Abstract

**Introduction:**

Microbes in the built environment have been implicated as a source of infectious diseases. Bacterial culture is the standard method for assessing the risk of exposure to pathogens in urban environments, but this method only accounts for <1% of the diversity of bacteria. Recently, full-length 16S rRNA gene analysis using nanopore sequencing has been applied for microbial evaluations, resulting in a rise in the development of long-read taxonomic tools for species-level classification. Regarding their comparative performance, there is, however, a lack of information.

**Methods:**

Here, we aim to analyze the concordance of the microbial community in the urban environment inferred by multiple taxonomic classifiers, including ARGpore2, Emu, Kraken2/Bracken and NanoCLUST, using our 16S-nanopore dataset generated by MegaBLAST, as well as assess their abilities to identify culturable species based on the conventional culture results.

**Results:**

According to our results, NanoCLUST was preferred for 16S microbial profiling because it had a high concordance of dominant species and a similar microbial profile to MegaBLAST, whereas Kraken2/Bracken, which had similar clustering results as NanoCLUST, was also desirable. Second, for culturable species identification, Emu with the highest accuracy (81.2%) and F1 score (29%) for the detection of culturable species was suggested.

**Discussion:**

In addition to generating datasets in complex communities for future benchmarking studies, our comprehensive evaluation of the taxonomic classifiers offers recommendations for ongoing microbial community research, particularly for complex communities using nanopore 16S rRNA sequencing.

## Introduction

Built environment refers to man-made or modified structures that provide people with living, working, and recreational spaces. With the development of urbanization, people spend most of their time indoors. Microorganisms, especially the *Enterococcus faecium*, *Staphylococcus aureus*, *Klebsiella pneumoniae*, *Acinetobacter baumannii, Pseudomonas aeruginosa*, and *Enterobacter* sp. (ESKAPE), in the built environment have become a common source of infection (Rice, 2008; Neiderud, 2015; Wang et al., 2022). Microbial profiling of high-touch surfaces is an effective way for the surveillance of infectious agents in public indoor environments (Danko et al., 2021).

While the conventional culture-based method effectively identifies microorganisms with anti-microbial resistance, it only reflects <1% of the bacterial diversity (Pedron et al., 2020; Sala-Comorera et al., 2020; Marshall et al., 2021). Next-generation sequencing (NGS) directly traces DNA from the urban samples and characterizes microbial communities in a higher resolution (Kerkhof, 2021). However, second-generation sequencing has a low phylogenetic resolution and could not accurately classify microorganisms below the genus level because of the limitation of short read length (Kerkhof, 2021). In recent years, nanopore sequencing, developed by Oxford Nanopore Technologies (ONTs), has become a dominant microbiome analysis platform, which could generate reads with lengths up to 2 × 10^6^ bases (Rodriguez-Perez et al., 2021). This enables bacterial identification to the species level with a 92–94% accuracy in mock communities (Benitez-Paez et al., 2016; Lao et al., 2022). Different bioinformatic pipelines have been established for the analysis of nanopore sequencing data.

To date, MegaBLAST is the gold-standard alignment tool for classifying nucleotide sequences, but it can be computationally intensive (Ye et al., 2019). The classification speed of the 5.7 million-read dataset was estimated to be 4 h using MegaBLAST, while a variety of alignment-free classifiers completed the task in significantly less time (<10 min) (Ye et al., 2019). Kraken2 coupled with Bracken is a recent alignment-free classifier that provides a fast taxonomic classification of nanopore sequence data (1 min) (Wood et al., 2019; Lu and Salzberg, 2020). It maintains high accuracy and sensitivity in metagenomics analysis by using the *k*-mer-based approach. After that, three long-read classifiers were developed. NanoCLUST, which is the first tailor-made method for full-length 16S amplicon sequencing of the nanopore, was published in 2021 (Rodriguez-Perez et al., 2021). It is based on Uniform Manifold Approximation and Projection (UMAP), followed by the construction of a polished read and subsequent BLAST classification. Nevertheless, it might provide false positives because of the use of consensus sequences (Curry et al., 2022). Recently, Emu has been published to profile microbial communities using full-length 16S data (Curry et al., 2022). It claimed that the implementation of an expectation–maximization (EM)-algorithm could identify bacterial abundance more accurately than all the existing methods. Meanwhile, ARGpore2 was established to identify antibiotic resistance genes (ARGs) and their host populations from nanopore reads (Cheng et al., 2022). It combined the results of Centrifuge and MetaPhlan2 (Metagenomic Phylogenetic Analysis) markergene database to annotate the taxonomy of nanopore reads (Segata et al., 2012; Kim et al., 2016).

While most researchers analyzed several mock communities of simple microbiomes to evaluate the accuracy, sensitivity, and specificity of the established classifiers (Deshpande et al., 2019; Winand et al., 2019; Leidenfrost et al., 2020; Urban et al., 2021), an actual and complex microbial community from the urban environment provides a better assessment of the classifiers. In this study, a total of 46 environmental samples were collected from the mass transit system of a city populated with 7.4 million people. The species-level microbial communities were determined by bacterial culture and nanopore 16S rRNA sequencing. Based on our nanopore dataset produced by MegaBLAST and conventional culture results, we aim to assess the clustering and concordance of the microbial community in the urban environment inferred by various taxonomic classifiers, namely ARGpore2, Kraken2/Bracken, NanoCLUST, and Emu. We demonstrated that the sequencing-based method uncovered misclassification and the absence of species, especially the pathogenic species, in the culture-based method. This study also offered an unbiased and comprehensive assessment of taxonomic classifiers for the analysis of full-length 16S reads in complex microbial communities, allowing researchers to identify the most reliable bioinformatic tools for the study of the urban microbiome.

## Materials and methods

### Sample collection

A total of 46 urban samples were collected from 18 stations of the mass transit system of Hong Kong in the period of July to October (summer season). Enviroscreen Sponges (Technical Service Consultants) were used to collect bacteria from the high-touch environmental surfaces, including handrails, ticket kiosks, automated teller machines (ATMs), elevators, and escalators according to the manufacturer’s protocol (Technical Service Consultants Ltd., 2014). The detail of the collection sites is shown in Supplementary Table S1. The sponges were filled with 50 mL of Milli-Q Water with 0.1% peptone water and 0.1% Tween 80 in a sealed bag and squeezed for 5 min. The extracts were collected and centrifuged for 15 min at 4,600 × *g*. The bacterial pellet was resuspended in 50 mL of peptone water and divided into two parts, culture (1 mL) and DNA extraction procedures (49 mL).

### Species identification by culture-based methods

The resuspended bacterial pellet was cultured using the Brain Heart Infusion Agar (BHI Agar, BD BBL™) and CHROMagar Extended Spectrum β-lactamases (ESBL, Kanto Chemical), carbapenem-resistant Enterobacteriaceae (CRE, Kanto Chemical), multi-drug-resistant Acinetobacter (MDRA, Kanto Chemical), methicillin-resistant *Staphylococcus Aureus* (MRSA, Kanto Chemical), and vancomycin-resistant Enterococcus (VRE, Kanto Chemical). A total of 100 μL of the resuspended bacterial pellet was poured over the agar plates and spread evenly using the plate spreader. Six culture plates were placed in the incubator for 24 h at 37°C for each sample. Isolates of different morphology were picked and identified at the species level (score, 2.00) by the IVD MALDI Biotyper Microflex^®^ with the database version BD-6763 (Bruker Daltonics, Bremen, Germany).

### DNA extraction

DNA was extracted from the bacterial suspension using the QIAamp BiOstic Bacteremia DNA Kit (Qiagen) according to the manufacturer’s protocol. In brief, DNA was released after bacterial cell lysis *via* mechanical homogenization. DNA was then purified by column-based methods after inhibitor removal procedures and collected with an elution buffer. The DNA content was then quantified using the Qubit 2.0 fluorometer (Thermo Fisher Scientific) with the double-stranded DNA (dsDNA) HS assay kit (Thermo Fisher Scientific).

### Taxonomic assignment by nanopore sequencing

Libraries were prepared using the 16S barcoding kit 1–24 (SQK-16S024) from ONT according to the manufacturer’s protocol and quantified using the Qubit as described earlier. A total of 24 barcoded libraries were then pooled with equal concentrations. After adapter ligation, sequencing was performed using the Flow Cell (R9.4.1) with the GridION (Oxford Nanopore Technologies) for >24 h. Raw reads from the nanopore sequencing were trimmed by NanoFilt (De Coster et al., 2018). Reads with an average read quality score of <8.0 and of length below 1,000 base pairs were discarded. The data details are listed in Supplementary Table S2. Urban samples with a total read of less than 10,000 were eliminated. The filtered clean reads were then classified by MegaBLAST (Nucleotide BLAST 2.13.0+) (Morgulis et al., 2008; Camacho et al., 2009), ARGpore2 (Cheng et al., 2022), Kraken2/Bracken (Kraken version 2.1.2 and Bracken 2.5) (Wood et al., 2019; Lu and Salzberg, 2020), Emu (emu v3.2.0) (Wang et al., 2022), and NanoCLUST (Rodriguez-Perez et al., 2021) using default parameters and database. In the MegaBLAST classification, the output of the first good hit found in the database with an E-value of less than 0.001 was chosen for each read (Shah et al., 2019). Species of relative abundance of <0.1% in the four classifiers were discarded in four classifiers (ARGpore2, Emu, Kraken2/Bracken, and NanoCLUST). Due to limited computer RAM, ARGpore2, Emu, and NanoCLUST were unable to analyze some of the samples. ARGpore2, Emu, and NanoCLUST were able to analyze 43, 44, and 22 of the 46 samples, respectively.

### Filtered 16S sequencing data processing

Microbial profiles of eight groups, namely ARGpore2, Emu, Kraken2/Bracken, NanoCLUST, MegaBLAST (46 sites), MegaBLAST_ARGpore2 (43 sites), MegaBLAST_Emu (44 sites), and MegaBLAST_NanoCLUST (22 sites), were generated by the average abundance of the overlapped species. Species present in only one classifier were eliminated. The mean abundance of MegaBLAST and the four classifiers was calculated by (no. of reads from each species/ total no. of reads × 100%) and (mean relative abundance/ no. of sites × 100%,) respectively. In parallel, 20 urban samples (Site4, Site5, Site7, Site11, Site12, Site18, Site20, Site21, Site23, Site24, Site26, Site29, Site31, Site32, Site35, Site40, Site41, Site43, Site45, and Site46) that could be analyzed by all four classifiers were selected for downstream clustering analysis.

### Principal component analysis and hierarchical clustering

For principal component analysis (PCA), the R function pcromp was used to generate the graph. Utilizing the 20 urban samples indicated earlier, a three-dimensional figure with the top three principal components (PC1, PC2, and PC3) was created by R using the microbial profiles of the five groups (ARGpore2, Emu, Kraken2/Bracken, NanoCLUST, and MegaBLAST). Afterward, hierarchical clustering was performed using the complete linkage method. The resulting species of dendrograms were separated into the earlier eight groups.

### Statistical analysis and data availability

The microbial communities were assessed using phyloseq coupled with R-based computational tools in R-studio to generate the graphs (McMurdie and Holmes, 2013; McMurdie and Holmes, 2014; McMurdie and Holmes, 2015; Callahan et al., 2016). Performances of the classifiers were calculated as Accuracy = (TP + TN)/(TP + FP + FN + TN), Precision = TP/(TP + FP), Sensitivity (Recall) = TP/(TP + FN), Specificity = TN/(TN + FP), and F1 score = 2 × (Recall × Precision) / (Recall + Precision); TP: true positive (species was found in both outcomes), TN: true negative (species was absent in both outcomes), FP: false positive (species was found in one of the outcomes), and FN: false negative (species was absent in one of the outcomes). The values were based on the species’ total concordance between MegaBLAST and the four classifiers, as well as the species’ total concordance between nanopore sequencing using various classifiers and conventional cultures. Sequence data were archived in the National Center for Biotechnology Information (NCBI) Short Read Archive (SRA) (PRJNA875407).

## Results

### Species-level taxonomic assignment of the urban samples using nanopore sequencing

**Figure 1:**
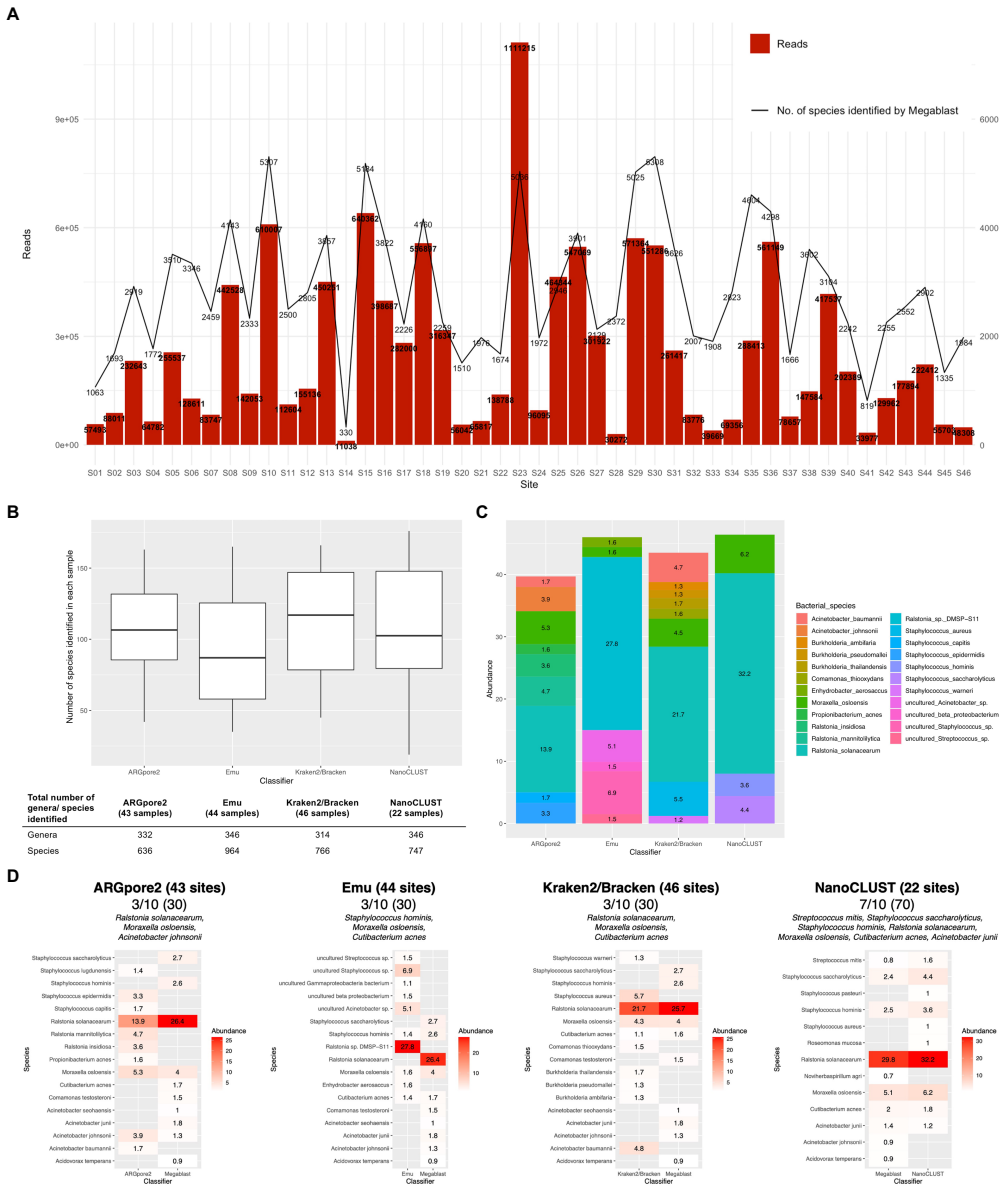
Species-level taxonomic assignment of the urban samples. **(A)** Total number of reads in each site and the number of species classified by MegaBLAST. **(B)** The number of bacterial species and genera in each sample identified by ARGpore2, Emu, Kraken2/Bracken, and NanoCLUST. **(C)** Average abundance of dominant species classified by ARGpore2, Emu, Kraken2/Bracken, and NanoCLUST. **(D)** Comparison of the top 10 dominant species between MegaBLAST and the four classifiers.

Collectively, 2,854 ± 1,250 species were identified in 46 urban samples by nanopore sequencing (256,112 ± 229,162 raw reads) using MegaBLAST (Figure 1A; Supplementary Table S3). Based on nanopore sequencing data from the same set of samples, Kraken2/Bracken was able to reveal the presence of 766 species and 314 genera (Supplementary Table S4) from 46 urban samples. Meanwhile, ARGpore2, Emu, and NanoCLUST analyses were performed but they could only provide a taxonomic classification for 43 (636 species and 332 genera, Supplementary Table S5), 44 (964 species and 346 genera, Supplementary Table S6), and 22 urban samples (747 species and 346 genera, Supplementary Table S7) because of limited computational resources (Figure 1B).

### Dominant species in each sample by the four classifiers

The overall microbiome profiles generated by the four classifiers are shown in Figure 1C (Supplementary Table S8). In ARGpore2 analysis, the most abundant species were *Ralstonia solanacearum* (13.9% ± 15.7%), *Moraxella osloensis* (5.3% ± 6.7%), *Ralstonia mannitolilytica* (4.7% ± 5.2%), and *Acinetobacter johnsonii* (3.9% ± 10.2%) (Figure 1C). For Emu, the dominant species included *Ralstonia* sp. *DMSP-S11* (27.8% ± 0.7%), *uncultured Staphylococcus* sp. (6.9% ± 0.1%), *uncultured Acinetobacter* sp. (5.1% ± 0.3%), and *Enhydrobacter aerosaccus* (1.6% ± 0.0%), while in Kraken2/Bracken classification, *Ralstonia solanacearum* (21.7% ± 24.2%) was also the most abundant, followed by *Staphylococcus aureus* (5.5% ± 4.0%), *Acinetobacter baumannii* (4.7% ± 7.3%), and *Moraxella osloensis* (4.5% ± 5.9%) (Figure 1C). It is important to note that *Enhydrobacter aerosaccus* was distinctively detectable in Emu. In addition, NanoCLUST generated a similar result, in which *Ralstonia solanacearum* (32.2% ± 28.8%), *Moraxella osloensis* (6.2% ± 7.6%), and *Staphylococcus saccharolyticus* (4.4% ± 3.4%) were dominant (Figure 1C). In addition, the concordance of the top 10 dominant species between MegaBLAST and the four classifiers was determined. NanoCLUST showed the highest concordance (7/10), followed by ARGpore2 (3/10), Emu (3/10), and Kraken2/Bracken (3/10) (Figure 1D). Among the concordant species, only *Moraxella osloensis* could be consistently identified by all classifiers, while *Ralstonia solanacearum* was identified by all classifiers except Emu (Figure 1D). *Cutibacterium acnes* could also be identified by all the classifiers except ARGpore2.

### Comparisons of the microbiomes classified by MegaBLAST and the four classifiers

**Figure 2:**
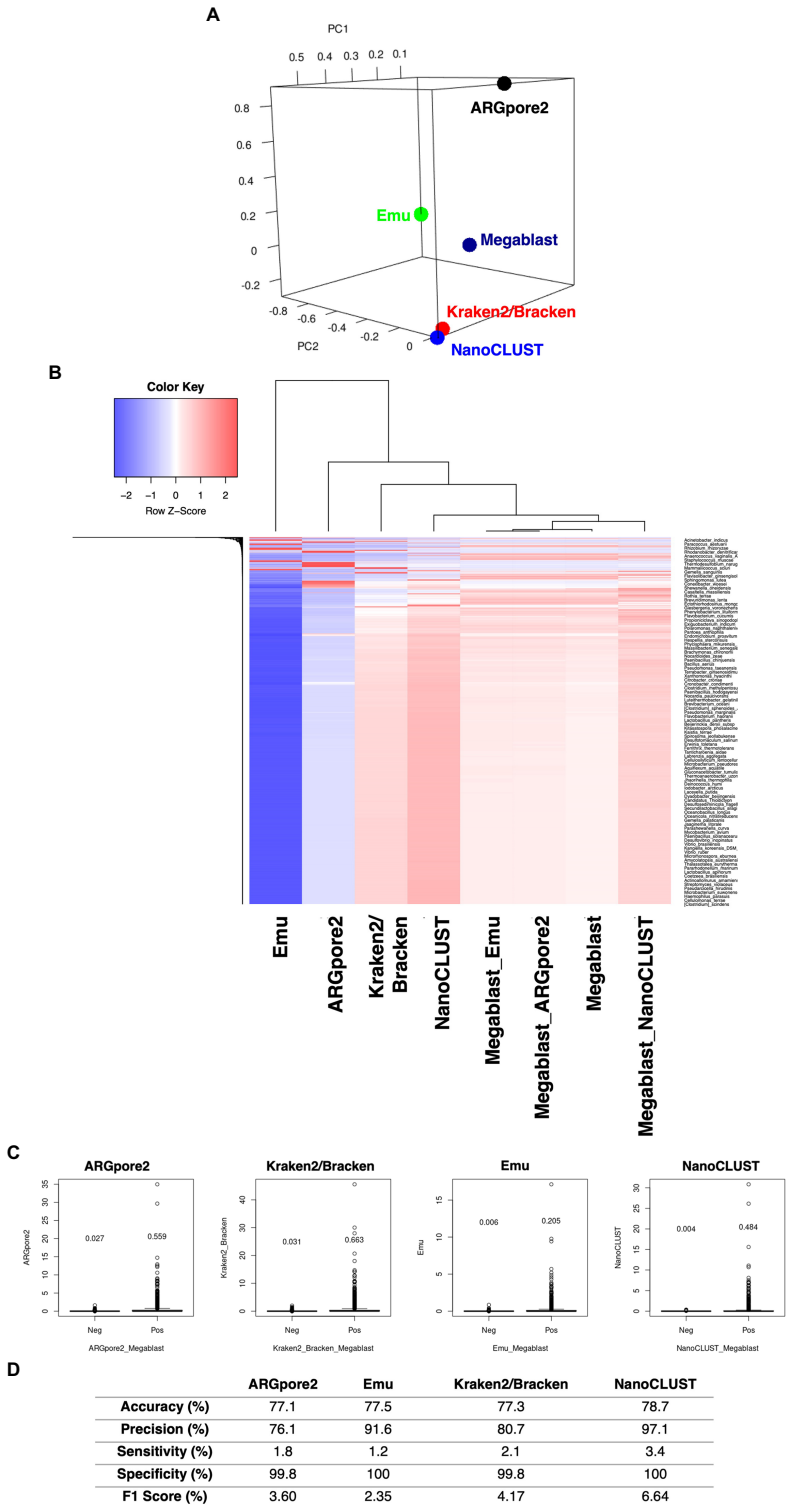
Comparisons of the microbiomes classified by MegaBLAST and the four classifiers. **(A)** Principal component analysis of microbiomes classified by MegaBLAST and the four classifiers, 20 urban examples that appeared in all of the classifiers were chosen. **(B)** Hierarchical clustering of the bacterial FIGURE 2 (Continued)species abundances classified by MegaBLAST, and the four classifiers **(C)** Species classified by ARGpore2, Emu, Kraken2/Bracken, and NanoCLUST were detectable (Pos) and undetectable (Neg) by MegaBLAST. The y-axis represents the abundance of the classified species, and the values were the abundance means. **(D)** The average values for accuracy, precision, sensitivity, specificity, and F1 score of the four classifiers based on the species detected by MegaBLAST.

In addition to the dominant core microbiome, microbial profiles classified by MegaBLAST and the four classifiers were also compared based on the abundance and microbial taxa results. In total, 20 urban samples from each of the four classifiers were chosen for principal component analysis (PCA) and hierarchical clustering analysis to ensure a fair comparison. PCA in Figure 2A illustrates that microbial profiles generated by the four classifiers were distinctively different except Kraken2/Bracken and NanoCLUST. Of them, the microbiome created by NanoCLUST (blue) and Kraken2/Bracken (red) had the highest similarity to that of MegaBLAST (dark blue) (Figure 2A; Supplementary Table S8). This observation was supported by the hierarchical clustering in Figure 2B (Supplementary Table S8), where the profile of NanoCLUST was clustered with MegaBLAST, followed by Kraken2/Bracken, ARGpore2, and Emu. Furthermore, the abundance of MegaBLAST-positive and negative species classified by ARGpore2, Emu, Kraken2/Bracken, and NanoCLUST is shown in Figure 2C. Importantly, the mean abundance of the detectable and undetectable species was 0.478% ± 0.196 and 0.017% ± 0.014%, respectively. Therefore, the 0.1% relative abundance cutoff was believed to be acceptable for filtering true negative species. The accuracy, precision, sensitivity, specificity, and F1 score of the four classifiers were then evaluated based on the species detected by MegaBLAST (Figure 2D; Supplementary Table S9). No significant differences were observed in the accuracy of the four classifiers but F1 scores showed the highest in NanoCLUST (6.64%), followed by Kraken2/Bracken (4.17%), ARGpore2 (3.60%), and Emu (2.35%) (Figure 2D).

### Comparisons of bacterial species identified by conventional culture and the four classifiers

**Figure 3:**
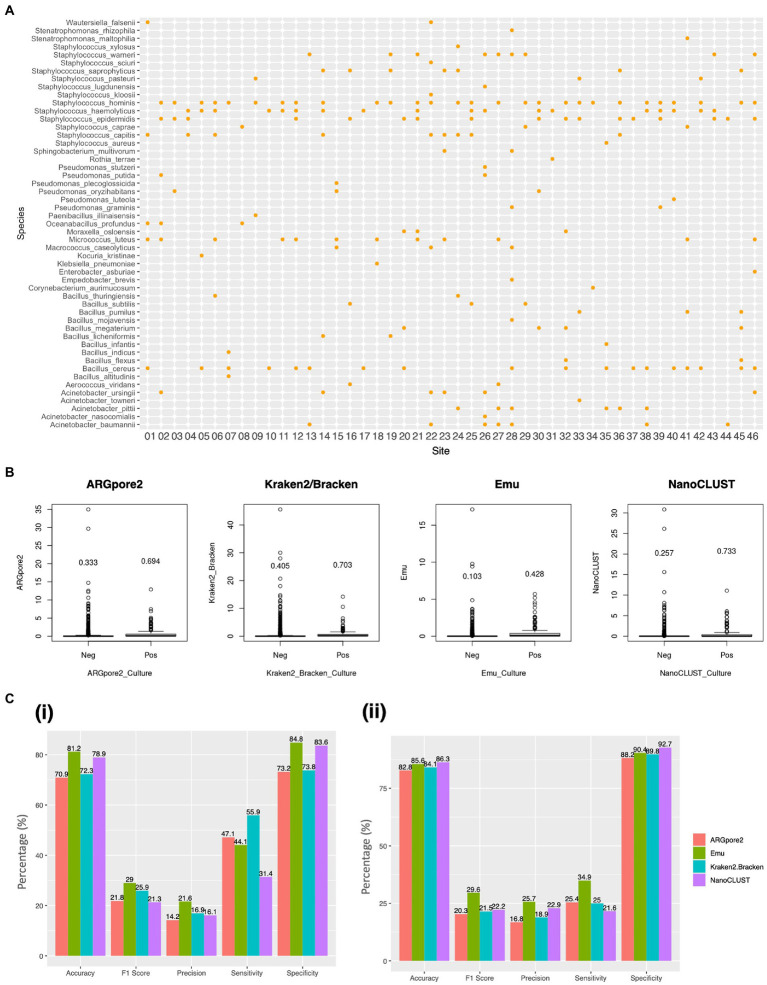
Comparisons of bacterial species identified by conventional culture and the four classifiers. **(A)** Bacterial presence in routine culture from the 46 urban samples. Each dot represents at least one colony that has been identified. **(B)** Species classified by ARGpore2, Emu, Kraken2/Bracken, and NanoCLUST were present (Pos) and absent (Neg) in culture. The y-axis represents the abundance of the classified species, and the values were the abundance means. **(C)** Bar plots of average accuracy, precision, sensitivity, specificity, and F1 score of the four classifiers based on the species present in culture using (i) 0.1% and (ii) mean abundance of the detectable species in MegaBLAST as the cutoff.

Overall, 51 species and 19 genera were identified in 46 urban samples by the culture-based method. The dominant species belonged to *Staphylococcus hominis* (*n* = 28), followed by *Bacillus cereus* (*n* = 18), *Staphylococcus haemolyticus* (*n* = 18), and *Staphylococcus epidermidis* (*n* = 17) (Figure 3A; Supplementary Table S10). The identified isolates are listed in Supplementary Table S10. Afterward, we found that the mean abundance of the culture-based detectable species classified by ARGpore2, Emu, Kraken2/Bracken, and NanoCLUST was 0.640% ± 0.142% (Figure 3B). Notably, the mean abundance of the culture-based undetectable was 0.275% ± 0.129%, indicating that the 0.1% relative abundance cutoff, as proposed in previous studies (Zaura et al., 2009; Doan et al., 2016; Sun et al., 2021), might not be sufficient to filter true negative species. The mean abundance of the detectable species in Emu was the lowest (0.428%), followed by ARGpore2 (0.694%), NanoCLUST (0.733%), and Kraken2/Bracken (0.703%) (Figure 3B). This might explain the best performance in Emu, which has the highest accuracy (81.2%), specificity (84.8%), and F1 score (29%) (Figure 3Ci). Meanwhile, the accuracy (78.9%) and specificity (83.6%) of NanoCLUST were as high as Emu (Figure 3Ci). Although Kraken2/Bracken has lower accuracy and specificity, its sensitivity (55.9%) was much higher than others (Figure 3Ci; Supplementary Table S11). When the cutoff was changed to the mean abundance of the detectable species in MegaBLAST, i.e., 0.559, 0.663, 0.205, and 0.484 for ARGpore2, Kraken2/Bracken, Emu, and NanoCLUST, respectively, the accuracy and the specificity of all the four classifiers were > 80% (Figure 3Cii; Supplementary Table S11). Notably, Emu has the highest sensitivity, while its F1 score was still the highest (29.6%) (Figure 3Cii).

### Nanopore 16S MegaBLAST failed to detect 13 culture-positive species

**Figure 4:**
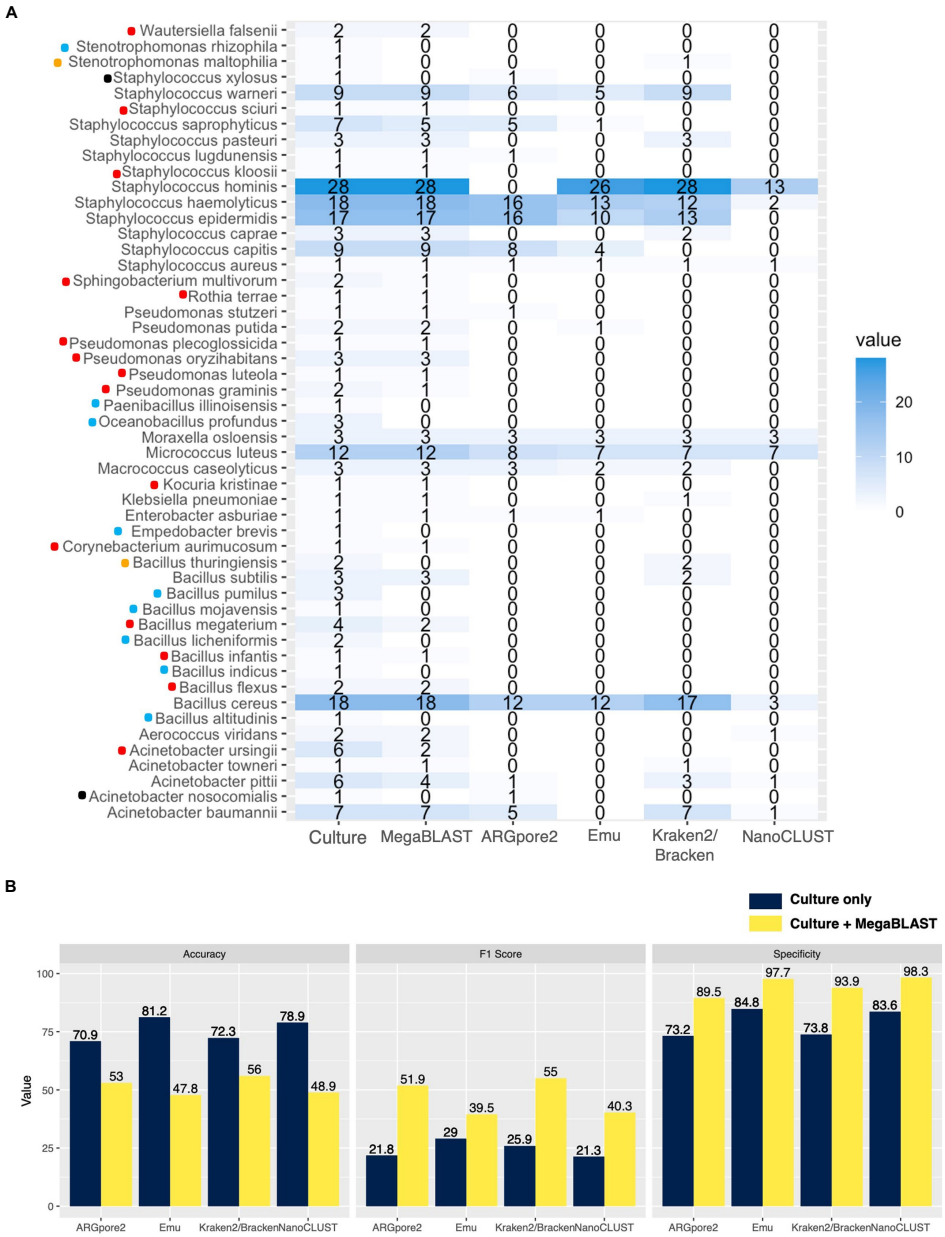
Concordance of the culturable species between MegaBLAST and the four classifiers. **(A)** Detectable and cultured species in the four classifiers. The value represents the number of urban samples with the corresponding bacterial species concordant with the culture results. Red dots indicate the detectable species in MegaBLAST, while blue dots represent the absence of the species in the sequencing data. Four species were not detected by MegaBLAST but could be identified by Kraken2/Bracken and ARGpore2, which were labeled with orange and black dots, respectively. **(B)** Average F1 score (%) and specificity (%) increased in the complement results of culture and MegaBLAST while accuracy (%) decreased.

Four species, *Acinetobacter nosocomialis, Bacillus thuringiensis, Staphylococcus xylosus,* and *Stenotrophomonas maltophilia,* were detected by Kraken2/Bracken (orange dots) or ARGpore2 (black dots) but not by MegaBLAST (Figure 4A). Another nine cultured species were undetectable in all classifiers (blue dots), with five of them being *Bacillus spp.* (Figure 4A). Two *Bacilli* (*Oceanobacillus profundus* and *Paenibacillus* illinoisensis), *Empedobacter brevis,* and *Stenotrophomonas rhizophila* were also undetectable (Figure 4A). Interestingly, *Empedobacter brevis* was found in the conventional culture of Site 28 only (Figure 3A), whereas *Empedobacter falsenii (Wautersiella falsenii)* was found in its sequencing data (Supplementary Table S3). Additionally, MegaBLAST was able to detect other *Oceanobacillus* species at Site 1, Site 2, and Site 8 (Supplementary Table S3), which harbored *Oceanobacillus profundus* in conventional cultures (Figure 3A). Taken together, these species might be misclassified using nanopore sequencing.

### Species that are culture- and MegaBLAST-positive but undetectable in the four classifiers

The number of cultured species detected by nanopore sequencing is shown in Figure 4A. Most of the cultured species (74.5%, 38/51) were detectable by MegaBLAST. Among the 38 species, 39.5% (15/38) could not be classified by the four classifiers, which are highlighted in red in Figure 4A, including *Wautersiella falsenii, Staphylococcus spps., Sphingobacterium multivorum, Rothia terrae, Pseudomonas* spp. *Kocuria kristinae, Corynebacterium aurimucosum, Bacillus spp.*, *and Acinetobacter ursingii.* For the detectable species (23/38, 60.5%), all classifiers could detect *Bacillus cereus, Micrococcus luteus, Moraxella osloensis, Staphylococcus aureus*, and *Staphylococcus haemolyticus* (Figure 4A). Importantly, only Kraken2/Bracken could detect *Bacillus subtilis, Klebsiella pneumoniae, Staphylococcus caprae*, and *Staphylococcus pasteuri* (Figure 4A). Although Emu could not detect *Acinetobacter* species because most of them are classified as *Acinetobacter* sp., it was the only classifier that can identify *Pseudomonas putida* and successfully found *Enterobacter asburiae, Staphylococcus capitis*, and *Staphylococcus saprophyticus*, while Kraken2/Bracken and NanoCLUST could not (Figure 4A). In addition, ARGpore2 exclusively detected *Pseudomonas stutzeri* and *Staphylococcus lugdunensis* (Figure 4A).

### Complementation of nanopore 16S MegaBLAST and conventional culture results

The concordance of the culture and the MegaBLAST results is shown in Supplementary Table S11. It was highlighted that 60.4% of the species present in the MegaBLAST results were uncultivated, which were considered false negatives in the culture. To eliminate false positives in the outputs of the four classifiers, the MegaBLAST data were combined with standard culture results to create a new set of the occurrences of these 51 bacterial species (Supplementary Table S12). After removing these false positives, F1 scores of the four classifiers increased from 24.5% ± 3.7 to 46.7% ± 7.9%, while specificity increased from 78.9% ± 6.2 to 94.9% ± 4.1% (Figure 4B; Supplementary Table S11). The accuracy, however, decreased from 75.8% ± 5.0 to 51.4% ± 3.8% (Figure 4B; Supplementary Table S11).

## Discussion

Herein we collected 46 samples from the mass transit system of Hong Kong and identified the bacterial community by conventional culture and nanopore full-length 16S rRNA sequencing. The performances of four taxonomic classifiers, namely ARGpore2, Emu, Kraken2/Bracken, and NanoCLUST, were evaluated based on a set of urban microbiome data generated by MegaBLAST. The ideal taxonomic classifier should demonstrate a high concordance of dominant species and a similar microbial profile as MegaBLAST, which was one of the most sensitive metagenomics alignment methods but computationally intensive (Ye et al., 2019). Second, we comprehensively compared the bacterial species identified by conventional culture and 16S nanopore sequencing and eventually provided recommendations for classifier selection.

Despite having a higher error rate and lower throughput than Illumina sequencing, nanopore sequencing can produce much longer reads, allowing for the study of complex microbial samples and the identification of rare taxa (Ciuffreda et al., 2021). Data analysis such as read mapping and *de novo* assembly for nanopore sequencing may be difficult due to specialized tools and expertise (Magi et al., 2018). Limited studies were performed to evaluate the performance of classifiers using 16S nanopore sequencing data (Ciuffreda et al., 2021; Curry et al., 2022). As such, our study looked into the use of nanopore sequencing for 16S microbial profiling. Kraken2/Bracken, which was not designed for nanopore sequencing, performed well in bacterial community classification, according to our findings. Following that, a comparison of Kraken2/Bracken to different nanopore classification tools (ARGpore2, Emu, and NanoCLUST) will be shown.

Previous studies demonstrated that the similarities between NanoCLUST/Emu and the expected mock communities were higher than that of Kraken2/Bracken (Rodriguez-Perez et al., 2021; Curry et al., 2022). While there were < 100 bacterial species in the mock communities used in NanoCLUST and Emu studies, our studies used actual communities with >700 distinct species. NanoCLUST, the first custom-built pipeline for analyzing nanopore 16S rRNA amplicon reads, had the highest concordance of dominant species and microbial profile similarity to MegaBLAST. In addition, NanoCLUST had the highest F1 score (6.64%). The underlying cause could be the use of subsequent BLAST classification in NanoCLUST (Rodriguez-Perez et al., 2021). Nevertheless, the time required for a 300,000-read dataset using NanoCLUST was 20 min, while MegaBLAST took >24 h in our computational analysis. In the clustering analysis, Kraken2/Bracken, which was the fastest taxonomic classifier (1 min) and could analyze all urban samples, had a similar microbial profile to NanoCLUST. Its F1 score (4.17%) was also higher than ARGpore2 (3.60%) and Emu (2.35%).

Curiously, the most prevalent species was *Ralstonia solanacearum* in all classifiers except Emu, where *Ralstonia* sp. *DMSP-S11* was the most abundant. In addition, Emu could not provide an exact *Acinetobacter* and *Staphylococcus* species and therefore reported hits at a higher taxonomic rank that was labeled as “*Acinetobacter sp.”* and “*Staphylococcus sp*.” Excluding the two species identified only at the genus level as “*Acinetobacter sp*.” and “*Staphylococcus sp*.”, there were still a hundred other species that were classified at the genus level. This might explain its distinct microbial profile when compared with MegaBLAST. Taken together, NanoCLUST was the most desirable classifier for complex community profiling, followed by Kraken2/Bracken, ARGpore2, and Emu.

In addition to microbial profiling, the performances in culturable species detection of the four classifiers were also estimated. Overall, the concordance between culture and 16S sequencing was low (38.6%). A previous study indicated the challenge of cutoff selection for accurate microbial identification in complex community analysis (Sun et al., 2021). The relative abundance of 0.1% as the typical cutoff to filter true negatives is still inconclusive. To that end, we used the mean abundance of the MegaBLAST-positive species as the cutoff and thus lead to overall increases in accuracy, F1 score, and specificity of the four classifiers. Of note, Emu, which could not provide accurate microbial profiling based on a set of urban microbiome data generated by MegaBLAST, has the highest F1 score in culturable species detection. The combination of rrnDB version 5.635 (Stoddard et al., 2015) and NCBI 16S RefSeq (O’Leary et al., 2016) in the Emu 16S database might benefit the species-level classification process and effectively reduce the number of unclassified or misclassified reads (Curry et al., 2021). Interestingly, only Emu could detect *Enhydrobacter aerosaccus* among the four classifiers, which was found in 41 urban samples. It was revealed that *Enhydrobacter aerosaccus* was enriched in the public transit air microbiome of Hong Kong (Leung et al., 2021), and *Enhydrobacter sp.* was more prevalent in Asian individuals (Leung et al., 2015). Furthermore, the ability to eliminate the misclassification caused by nanopore sequencing through the expectation–maximization algorithm in Emu might explain the additional concordant species classified by Emu (Curry et al., 2021). Hence, the distinct microbial profiling by Emu is caused by the uncertainty of species-level identification. Only genus-level identification was given for *Staphylococcus sp.* and *Acinetobacter sp.* Therefore, Emu would be preferable if the uncertainty of species identification is improved. The inability of species-level classification, in addition to Emu, may account for the discrepancy between culture and 16S sequencing for all classifiers.

In the in-depth analysis of the concordance between culture and nanopore sequencing, 17.6% of culturable species were undetectable. Previous studies suggested that DNA isolation procedures, the main determinants of sequencing results, could result in some degree of DNA loss that might lower the final DNA quantities of some bacterial species and eventually cause undetectable species (Knudsen et al., 2016). Furthermore, misclassification is still a challenge to classify genomes from distinct species with a high genomic identity (Wood et al., 2019), especially for *Bacillus* spp. in our results. It has been indicated that *Bacillus cereus* was misclassified as *Bacillus thuringiensis,* owing to the 99.73% similarity in their 16S rRNA sequence (Rodriguez-Perez et al., 2021). Furthermore, MegaBLAST might misclassify *Empedobacter brevis* as *Empedobacter falsenii* (*Wautersiella falsenii*) due to its highest 16S rRNA gene sequence similarity (Schauss et al., 2015). Overall, the mismatch between culture and 16S sequencing could also be explained by the loss of bacterial DNA and misclassification.

Our results demonstrated that bacterial species of the highest abundance (*Ralstonia solanacearum*) in 16S sequencing were not present in the culture. Typically, 48–72 h of incubation at 28°C instead of 37°C is required for *R. solanacearum* (Pawaskar et al., 2014). Many bacterial species are unculturable in general growth conditions or survive before sample processing (Tringe and Rubin, 2005). In addition, it was reported that uncultured genera comprise 81% of bacterial species across urban environments (Lloyd et al., 2018). Meanwhile, some bacterial species may be missed in MALDI identification, leading to false negative results in conventional culture. The findings of combining MegaBLAST with conventional culture results showed that culture-negative but MegaBLAST-positive results significantly influenced the F1 scores of the four classifiers.

To conclude, it is suggested to use various tools depending on the applications. For 16S microbial profiling, NanoCLUST with a high concordance of dominant species and a similar microbial profile to MegaBLAST was desirable whereas Kraken2/Bracken, which had comparable clustering results to NanoCLUST, was also recommended. Second, Emu was preferred for recognizing culturable species accurately. Our unbiased and unsupervised assessment strategy could comprehensively evaluate newly developed classifiers, improved classifiers, and established classifiers of different step parameters for complex communities. It is recommended that the performance of the classifiers be investigated for both simple and complex communities. When using these classifiers, it will be beneficial to provide different parameters or settings, such as community complexity and sample types.

## Data availability statement

The datasets presented in this study can be found in online repositories. The names of the repository/repositories and accession number(s) can be found in the article/Supplementary material.

## Author contributions

AL, CC, and GS conceived the original concept and designed the study. AL, CC, LW, C-YY, W-TL, and K-CC performed the experiments. AL analyzed the data and wrote the original manuscript. GS and AL amended and revised the writing. All authors read and approved the final version of the manuscript.

## Funding

This manuscript was supported by Interdisciplinary Large External Project Application Scheme from the Faculty of Health and Social Sciences, The Hong Kong Polytechnic University (1-ZVZL).

## Conflict of interest

The authors declare that the research was conducted in the absence of any commercial or financial relationships that could be construed as a potential conflict of interest.

## Publisher’s note

All claims expressed in this article are solely those of the authors and do not necessarily represent those of their affiliated organizations, or those of the publisher, the editors and the reviewers. Any product that may be evaluated in this article, or claim that may be made by its manufacturer, is not guaranteed or endorsed by the publisher.

## References

[ref1] Benitez-Paez A. Portune K. J. Sanz Y. (2016). Species-level resolution of 16S rRNA gene amplicons sequenced through the MinION portable nanopore sequencer. Gigascience 5:4. doi: 10.1186/s13742-016-0111-z, PMID: 26823973 PMC4730766

[ref2] Callahan B. J. Sankaran K. Fukuyama J. A. McMurdie P. J. Holmes S. P. (2016). Bioconductor workflow for microbiome data analysis: from raw reads to community analyses. F1000Res 5:1492. doi: 10.12688/f1000research.8986.2, PMID: 27508062 PMC4955027

[ref3] Camacho C. Coulouris G. Avagyan V. Ma N. Papadopoulos J. Bealer K.. (2009). BLAST+: architecture and applications. BMC Bioinformat. 10:421. doi: 10.1186/1471-2105-10-421, PMID: 20003500 PMC2803857

[ref4] Cheng H. Sun Y. Yang Q. Deng M. Yu Z. Liu L.. (2022). An ultra-sensitive bacterial pathogen and antimicrobial resistance diagnosis workflow using Oxford Nanopore adaptive sampling sequencing method. medRxiv. doi: 10.1101/2022.07.03.22277093 36259361

[ref5] Ciuffreda L. Rodriguez-Perez H. Flores C. (2021). Nanopore sequencing and its application to the study of microbial communities. Comput. Struct. Biotechnol. J. 19, 1497–1511. doi: 10.1016/j.csbj.2021.02.020, PMID: 33815688 PMC7985215

[ref6] Curry K. D. Wang Q. Nute M. G. Tyshaieva A. Reeves E. Soriano S.. (2021). Emu: Species-level microbial community profiling for full-length Nanopore 16S reads. bioRxiv. doi: 10.1101/2021.05.02.442339 PMC9939874 35773532

[ref7] Curry K. D. Wang Q. Nute M. G. Tyshaieva A. Reeves E. Soriano S.. (2022). Emu: species-level microbial community profiling of full-length 16S rRNA Oxford Nanopore sequencing data. Nat. Methods 19, 845–853. doi: 10.1038/s41592-022-01520-4, PMID: 35773532 PMC9939874

[ref8] Danko D. Bezdan D. Afshin E. E. Ahsanuddin S. Bhattacharya C. Butler D. J.. (2021). A global metagenomic map of urban microbiomes and antimicrobial resistance. Cells 184, 3376–3393.e17 e3317. doi: 10.1016/j.cell.2021.05.002, PMID: 34043940 PMC8238498

[ref9] De Coster W. D'Hert S. Schultz D. T. Cruts M. Van Broeckhoven C. (2018). NanoPack: visualizing and processing long-read sequencing data. Bioinformatics 34, 2666–2669. doi: 10.1093/bioinformatics/bty149, PMID: 29547981 PMC6061794

[ref10] Deshpande S. V. Reed T. M. Sullivan R. F. Kerkhof L. J. Beigel K. M. Wade M. M. (2019). Offline next generation Metagenomics sequence analysis using MinION detection software (MINDS). Genes (Basel) 10:578. doi: 10.3390/genes10080578, PMID: 31366182 PMC6723491

[ref11] Doan T. Akileswaran L. Andersen D. Johnson B. Ko N. Shrestha A.. (2016). Paucibacterial microbiome and resident DNA virome of the healthy conjunctiva. Invest. Ophthalmol. Vis. Sci. 57, 5116–5126. doi: 10.1167/iovs.16-19803, PMID: 27699405 PMC5054734

[ref12] Kerkhof L. J. (2021). Is Oxford Nanopore sequencing ready for analyzing complex microbiomes? FEMS Microbiol. Ecol. 97:fiab001. doi: 10.1093/femsec/fiab001, PMID: 33444433 PMC8068755

[ref13] Kim D. Song L. Breitwieser F. P. Salzberg S. L. (2016). Centrifuge: rapid and sensitive classification of metagenomic sequences. Genome Res. 26, 1721–1729. doi: 10.1101/gr.210641.116, PMID: 27852649 PMC5131823

[ref14] Knudsen B. E. Bergmark L. Munk P. Lukjancenko O. Prieme A. Aarestrup F. M.. (2016). Impact of sample type and DNA isolation procedure on genomic inference of microbiome composition. mSystems 1:16. doi: 10.1128/mSystems.00095-16, PMID: 27822556 PMC5080404

[ref15] Lao H. Y. Ng T. T. Wong R. Y. Wong C. S. Lee L. K. Wong D. S.. (2022). The clinical utility of two high-throughput 16S rRNA gene sequencing workflows for taxonomic assignment of unidentifiable bacterial pathogens in matrix-assisted laser desorption ionization-time of flight mass spectrometry. J. Clin. Microbiol. 60:e0176921. doi: 10.1128/JCM.01769-21, PMID: 34788113 PMC8769742

[ref16] Leidenfrost R. M. Pöther D.-C. Jäckel U. Wünschiers R. (2020). Benchmarking the MinION: evaluating long reads for microbial profiling. Sci. Rep. 10:5125. doi: 10.1038/s41598-020-61989-x, PMID: 32198413 PMC7083898

[ref17] Leung M. H. Y. Tong X. Boifot K. O. Bezdan D. Butler D. J. Danko D. C.. (2021). Characterization of the public transit air microbiome and resistome reveals geographical specificity. Microbiome 9:112. doi: 10.1186/s40168-021-01044-7, PMID: 34039416 PMC8157753

[ref18] Leung M. H. Wilkins D. Lee P. K. (2015). Insights into the pan-microbiome: skin microbial communities of Chinese individuals differ from other racial groups. Sci. Rep. 5:11845. doi: 10.1038/srep11845, PMID: 26177982 PMC4503953

[ref19] Lloyd K. G. Steen A. D. Ladau J. Yin J. Crosby L. (2018). Phylogenetically novel uncultured microbial cells dominate earth microbiomes. mSystems 3:18. doi: 10.1128/mSystems.00055-18, PMID: 30273414 PMC6156271

[ref20] Lu J. Salzberg S. L. (2020). Ultrafast and accurate 16S rRNA microbial community analysis using kraken 2. Microbiome 8:124. doi: 10.1186/s40168-020-00900-2, PMID: 32859275 PMC7455996

[ref21] Magi A. Semeraro R. Mingrino A. Giusti B. D’Aurizio R. (2018). Nanopore sequencing data analysis: state of the art, applications and challenges. Brief. Bioinform. 19, 1256–1272. doi: 10.1093/bib/bbx062, PMID: 28637243

[ref22] Marshall C. W. Kurs-Lasky M. McElheny C. L. Bridwell S. Liu H. Shaikh N. (2021). Performance of conventional urine culture compared to 16S rRNA gene amplicon sequencing in children with suspected urinary tract infection. Microbiol Spectr 9:e0186121. doi: 10.1128/spectrum.01861-21, PMID: 34937185 PMC8694219

[ref23] McMurdie P. J. Holmes S. (2013). Phyloseq: an R package for reproducible interactive analysis and graphics of microbiome census data. PLoS One 8:e61217. doi: 10.1371/journal.pone.0061217, PMID: 23630581 PMC3632530

[ref24] McMurdie P. J. Holmes S. (2014). Waste not, want not: why rarefying microbiome data is inadmissible. PLoS Comput. Biol. 10:e1003531. doi: 10.1371/journal.pcbi.1003531, PMID: 24699258 PMC3974642

[ref25] McMurdie P. J. Holmes S. (2015). Shiny-phyloseq: web application for interactive microbiome analysis with provenance tracking. Bioinformatics 31, 282–283. doi: 10.1093/bioinformatics/btu616, PMID: 25262154 PMC4287943

[ref26] Morgulis A. Coulouris G. Raytselis Y. Madden T. L. Agarwala R. Schaffer A. A. (2008). Database indexing for production MegaBLAST searches. Bioinformatics 24, 1757–1764. doi: 10.1093/bioinformatics/btn322, PMID: 18567917 PMC2696921

[ref27] Neiderud C. J. (2015). How urbanization affects the epidemiology of emerging infectious diseases. Infect Ecol Epidemiol 5:27060. doi: 10.3402/iee.v5.27060 26112265 PMC4481042

[ref28] O’Leary N. A. Wright M. W. Brister J. R. Ciufo S. Haddad D. McVeigh R.. (2016). Reference sequence (RefSeq) database at NCBI: current status, taxonomic expansion, and functional annotation. Nucleic Acids Res. 44, D733–D745. doi: 10.1093/nar/gkv1189, PMID: 26553804 PMC4702849

[ref29] Pawaskar J. Joshi M. Navathe S. Agale R. (2014). Physiological and biochemical characters of Ralstonia solanacearum. Int. J. Res. Agricult. Sci. 1, 2348–3997.

[ref30] Pedron J. Guyon L. Lecomte A. Blottiere L. Chandeysson C. Rochelle-Newall E.. (2020). Comparison of environmental and culture-derived bacterial communities through 16S Metabarcoding: a powerful tool to assess media selectivity and detect rare taxa. Microorganisms 8:1129. doi: 10.3390/microorganisms8081129, PMID: 32727027 PMC7464939

[ref31] Rice L. B. (2008). Federal funding for the study of antimicrobial resistance in nosocomial pathogens: no ESKAPE. J. Infect. Dis. 197, 1079–1081. doi: 10.1086/533452, PMID: 18419525

[ref32] Rodriguez-Perez H. Ciuffreda L. Flores C. (2021). NanoCLUST: a species-level analysis of 16S rRNA nanopore sequencing data. Bioinformatics 37, 1600–1601. doi: 10.1093/bioinformatics/btaa900, PMID: 33079990

[ref33] Sala-Comorera L. Caudet-Segarra L. Galofre B. Lucena F. Blanch A. R. Garcia-Aljaro C. (2020). Unravelling the composition of tap and mineral water microbiota: divergences between next-generation sequencing techniques and culture-based methods. Int. J. Food Microbiol. 334:108850. doi: 10.1016/j.ijfoodmicro.2020.108850, PMID: 32919261

[ref34] Schauss T. Busse H. J. Golke J. Kampfer P. Glaeser S. P. (2015). Empedobacter stercoris sp. nov., isolated from an input sample of a biogas plant. Int. J. Syst. Evol. Microbiol. 65, 3746–3753. doi: 10.1099/ijsem.0.000486, PMID: 26228269

[ref35] Segata N. Waldron L. Ballarini A. Narasimhan V. Jousson O. Huttenhower C. (2012). Metagenomic microbial community profiling using unique clade-specific marker genes. Nat. Methods 9, 811–814. doi: 10.1038/nmeth.2066, PMID: 22688413 PMC3443552

[ref36] Shah N. Nute M. G. Warnow T. Pop M. (2019). Misunderstood parameter of NCBI BLAST impacts the correctness of bioinformatics workflows. Bioinformatics 35, 1613–1614. doi: 10.1093/bioinformatics/bty833, PMID: 30247621

[ref37] Stoddard S. F. Smith B. J. Hein R. Roller B. R. Schmidt T. M. (2015). rrnDB: improved tools for interpreting rRNA gene abundance in bacteria and archaea and a new foundation for future development. Nucleic Acids Res. 43, D593–D598. doi: 10.1093/nar/gku1201, PMID: 25414355 PMC4383981

[ref38] Sun Z. Huang S. Zhang M. Zhu Q. Haiminen N. Carrieri A. P.. (2021). Challenges in benchmarking metagenomic profilers. Nat. Methods 18, 618–626. doi: 10.1038/s41592-021-01141-3, PMID: 33986544 PMC8184642

[ref01] Technical Service Consultants Ltd (2014). INSTRUCTION FOR USE Hygiene Sponge with Neutraliser. Biotrading Available at: http://biotrading.com/assets/productinformatie/tsc/ifu/ifu_ts15b.pdf

[ref39] Tringe S. G. Rubin E. M. (2005). Metagenomics: DNA sequencing of environmental samples. Nat. Rev. Genet. 6, 805–814. doi: 10.1038/nrg1709 16304596

[ref40] Urban L. Holzer A. Baronas J. J. Hall M. B. Braeuninger-Weimer P. Scherm M. J.. (2021). Freshwater monitoring by nanopore sequencing. elife 10:61504. doi: 10.7554/eLife.61504, PMID: 33461660 PMC7815314

[ref41] Wang H. Liu N. Chen J. Guo S. (2022). The relationship between Urban renewal and the built environment: a systematic review and Bibliometric analysis. J. Plan. Lit. 37, 293–308. doi: 10.1177/08854122211058909

[ref42] Winand R. Bogaerts B. Hoffman S. Lefevre L. Delvoye M. Braekel J. V.. (2019). Targeting the 16s Rrna gene for bacterial identification in complex mixed samples: comparative evaluation of second (Illumina) and third (Oxford Nanopore technologies) generation sequencing technologies. Int. J. Mol. Sci. 21:298. doi: 10.3390/ijms21010298, PMID: 31906254 PMC6982111

[ref43] Wood D. E. Lu J. Langmead B. (2019). Improved metagenomic analysis with kraken 2. Genome Biol. 20:257. doi: 10.1186/s13059-019-1891-0, PMID: 31779668 PMC6883579

[ref44] Ye S. H. Siddle K. J. Park D. J. Sabeti P. C. (2019). Benchmarking Metagenomics tools for taxonomic classification. Cells 178, 779–794. doi: 10.1016/j.cell.2019.07.010, PMID: 31398336 PMC6716367

[ref45] Zaura E. Keijser B. J. F. Huse S. M. Crielaard W. (2009). Defining the healthy" core microbiome" of oral microbial communities. BMC Microbiol. 9, 1–12. doi: 10.1186/1471-2180-9-259 20003481 PMC2805672

